# Left atrial reservoir strain improves diagnostic accuracy of the 2016 ASE/EACVI diastolic algorithm in patients with preserved left ventricular ejection fraction: insights from the KARUM haemodynamic database^[Author-notes jeac036-FM1]^

**DOI:** 10.1093/ehjci/jeac036

**Published:** 2022-02-19

**Authors:** Ashwin Venkateshvaran, Hande Oktay Tureli, Ulrika Ljung Faxén, Lars H Lund, Erik Tossavainen, Per Lindqvist

**Affiliations:** Department of Medicine, Cardiology Unit, Karolinska Institutet, D1:04, Eugeniavagen 3, Solna 171 64, Sweden; Department of Clinical Physiology, Surgical and Perioperative sciences, Umeå University, Universitetstorget 4, Umeå 901 87, Sweden; Department of Medicine, Cardiology Unit, Karolinska Institutet, D1:04, Eugeniavagen 3, Solna 171 64, Sweden; Department of Perioperative Medicine and Intensive Care, Norrbacka S2:05, Karolinska University Hospital, Solna 171 64, Sweden; Department of Medicine, Cardiology Unit, Karolinska Institutet, D1:04, Eugeniavagen 3, Solna 171 64, Sweden; Department of Cardiology, Public Health and Clinical Medicine, Umeå University, Universitetstorget 4, Umeå 901 87, Sweden; Department of Clinical Physiology, Surgical and Perioperative sciences, Umeå University, Universitetstorget 4, Umeå 901 87, Sweden

**Keywords:** left ventricular filling pressure, echo Doppler, right heart catheterization, speckle tracking echocardiography, diastolic dysfunction

## Abstract

**Aims:**

This study aimed to investigate the incremental value offered by left atrial reservoir strain (LAS_r_) to the 2016 American Society of Echocardiography/European Association of Cardiovascular Imaging (ASE/EACVI) diastolic algorithm to identify elevated left ventricular (LV) filling pressure in patients with preserved ejection fraction (EF).

**Methods and results:**

Near-simultaneous echocardiography and right heart catheterization were performed in 210 patients with EF ≥50% in a large, dual-centre study. Elevated filling pressure was defined as invasive pulmonary capillary wedge pressure (PCWP) ≥15 mmHg. LAS_r_ was evaluated using speckle-tracking echocardiography. Diagnostic performance of the ASE/EACVI diastolic algorithm was validated against invasive reference and compared with modified algorithms incorporating LAS_r_. Modest correlation was observed between *E*/*e*′, *E*/*A* ratio, and LA volume index with PCWP (*r* = 0.46, 0.46, and 0.36, respectively; *P* < 0.001 for all). Mitral *e*′ and TR peak velocity showed no association. The ASE/EACVI algorithm (89% feasibility, 71% sensitivity, 68% specificity) demonstrated reasonable ability (AUC = 0.69) and 68% accuracy to identify elevated LV filling pressure. LAS_r_ displayed strong ability to identify elevated PCWP (AUC = 0.76). Substituting TR peak velocity for LAS_r_ in the algorithm (69% sensitivity, 84% specificity) resulted in 91% feasibility, 81% accuracy, and stronger agreement with invasive measurements. Employing LAS_r_ as per expert consensus (71% sensitivity, 70% specificity) and adding LAS_r_ to conventional parameters (67% sensitivity, 84% specificity) also demonstrated greater feasibility (98% and 90%, respectively) and overall accuracy (70% and 80%, respectively) to estimate elevated PCWP.

**Conclusions:**

LAS_r_ improves feasibility and overall accuracy of the ASE/EACVI algorithm to discern elevated filling pressures in patients with preserved EF.

**See the editorial comment for this article ‘Left atrial strain imaging: ready for clinical implementation in heart failure with preserved ejection fraction’, by Espen Boe and Otto A. Smiseth, https://doi.org/10.1093/ehjci/jeac059.**

## Introduction

Chronic dyspnoea and fatigue are common symptoms in heart failure (HF) and pose a significant diagnostic challenge. These typical HF symptoms are often non-specific and lack accuracy to be independently employed for diagnosis, necessitating additional investigations.^1^ Doppler echocardiography is integral to routine evaluation in the setting of suspected HF and provides vital information on cardiac size, left ventricular (LV) performance, and valvular function. In the setting of HF with preserved ejection fraction (EF) (HFpEF), Doppler imaging combined with 2D echocardiography provides information on LV filling pressure critical to diagnosis and optimal therapy regulation.

In 2016, the American Society of Echocardiography (ASE) and European Association of Cardiovascular Imaging (EACVI) introduced an algorithm incorporating multiple common echocardiographic variables to ascertain LV filling pressure status.^2^ Studies evaluating the diagnostic accuracy of this algorithm in patients with normal EF, however, are limited and suggest poor or modest performance.^3–6^ Left atrial reservoir strain (LAS_r_) has been proposed as a novel, non-invasive diagnostic for grading diastolic dysfunction severity in patients with preserved EF.^7^ In a recent study, Inoue *et al.*^8^ demonstrated that LAS_r_ and LA pump strain demonstrate stronger ability to identify elevated filling pressures than conventional echocardiographic measures in a large HF population. However, LAS_r_ displayed generally poorer diagnostic performance in patients with preserved when compared with reduced LV systolic function in this study. Subsequently, the EACVI recommendations for multimodality imaging in HFpEF have suggested that incorporating LAS_r_ into the ASE/EACVI diagnostic algorithm may improve its diagnostic performance.^9^

With this background, we examined the additional diagnostic value offered by LAS_r_ to the 2016 ASE/EACVI diastolic algorithm in a large, dual-centre, haemodynamic database of patients with unexplained dyspnoea, and preserved LV EF employing invasive Pulmonary capillary wedge pressure (PCWP) as reference.

## Methods

### Study population

We retrospectively analysed all patients enrolled in the KARUM study, a dual-centre database of patients with unexplained dyspnoea undergoing near-simultaneous echocardiography and right heart catheterization (RHC). Details of the total KARUM cohort have been previously published.^10^ Patients were evaluated at two referral centres in Sweden; Karolinska University Hospital in Stockholm between 2014 and 2018, and Norrlands University Hospital in Umeå between 2010 and 2015. All subjects were haemodynamically stable during assessment. Prior to inclusion, we excluded patients with acute coronary syndrome, valvular prosthesis, and left bundle branch block. Thereafter, we excluded specific conditions where diastolic function assessment using the ASE/EACVI algorithm has significant limitations.^2^ This included patients with atrial fibrillation, pacemaker, hypertrophic or restrictive cardiomyopathy, and those that had undergone a heart transplantation or presented with >mild concomitant mitral valve disease. Finally, we excluded patients with EF <50% or poor echocardiographic image quality. Study protocol was approved by the local ethics committee at each centre (Karolinska: DNR 2008/1695-31 and Norrland: 07-092M, 2014-198-32M, and 2017-102-32M). All patients provided written informed consent.

### Echocardiography

Comprehensive echocardiography was performed in both centres by experienced echocardiographers (A.V. and P.L.) employing commercial ultrasound systems (Vivid E9, GE Ultrasound, Horten, Norway) in keeping with current recommendations.^11^ Pharmacological status was unaltered between echocardiography and catheterization. Digital loops were stored and analysed offline (EchoPAC PC, version 11.0.0.0 GE Ultrasound, Waukesha, Wisconsin) by experts (A.V. and P.L.) blinded to catheterization data. Mitral inflow interrogation was performed using pulsed-wave (PW) Doppler in the apical four-chamber view, placing the sample volume at the mitral leaflet tips. Transmitral early (*E*) and late diastolic velocities (*A*) were obtained and mitral *E*/*A* ratio computed. Doppler tissue imaging was utilized to measure early mitral annular velocities (*e*′) at both septal and lateral walls and subsequently averaged to calculate *E*/*e*′_mean_. Maximal tricuspid regurgitation (TR) peak velocity was measured using continuous-wave (CW) Doppler. Left atrial (LA) volume was obtained from apical four- and two-chamber views and indexed for body surface area.

**Figure 1:**
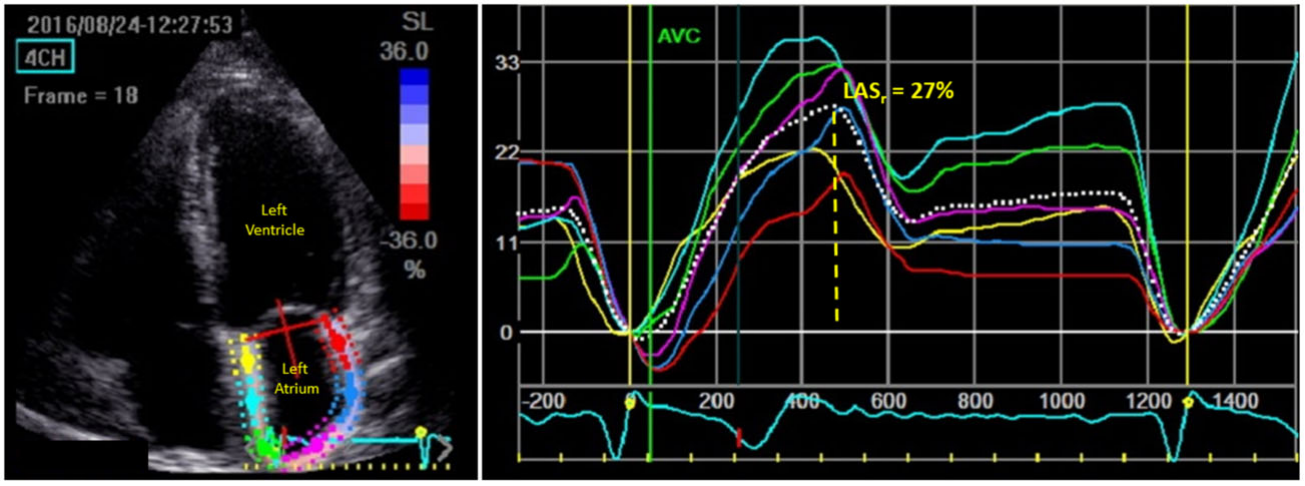
Apical four-chamber view illustrating speckle-tracking-enabled tracing and the corresponding strain curve.

Assessment of diastolic function and filling pressure status was performed employing the recommended two-step ASE/EACVI approach.^2^ In Step 1, diastolic function was evaluated considering recommended cut-offs provided for *e*′ (septal and/or lateral), *E*/*e*′_mean_, TR peak velocity, and indexed LA volume and graded as normal (where <50% of the four echocardiographic parameters were positive), indeterminate (50% positive), or definite diastolic dysfunction (>50% positive). In Step 2, filling pressures were assessed as normal, indeterminate, or elevated among patients with diastolic dysfunction employing transmitral *E*-velocity and *E*/*A* ratio and supplemented by *E*/*e*′_mean_, TR peak velocity and indexed LA as recommended.^2^ LAS_r_ was assessed using 2D speckle tracking echocardiography in keeping with expert recommendation.^12^ In a non-foreshortened apical four-chamber view, the LA endocardial border was traced, taking care to exclude the appendage and pulmonary veins. The region of interest (ROI) was then visually inspected for quality of tracking, repeated if found inadequate and excluded if still suboptimal. Zero point was set at the onset of the QRS complex on the ECG. LAS_r_ was defined as the maximal inflection point above the baseline during ventricular systole and assigned a positive value. Acquisition of LAS_r_ using speckle-tracking echocardiography is illustrated in *Figure 1*.

### Right heart catheterization

RHC was performed immediately after echocardiography (within a 3-h period) during the same visit by experts blinded to imaging results at each centre using fluid-filled 6F Swan–Ganz catheters employing jugular or femoral vein access. Transducers were zeroed at mid-thoracic level in each patient. PCWP was measured at mid-*A* wave and averaged from a minimum of five heart cardiac cycles. All pressure tracings were digitally stored and analysed offline using standard haemodynamic software package (WITT Series III, Witt Biomedical Corp., Melbourne, FL, USA).

### Reproducibility analysis

Intra- and inter-observer variability analysis for LAS_r_ was performed in 40 randomly selected patients. Measurements were performed first by the same operator on two subsequent days considering the same heart cycle, and then by independent readers blinded to each other in addition to invasive pressure readings. Measurement variability was expressed in coefficient of variation and intra-class coefficients.

### Statistical analysis

Normality was tested using the Shapiro–Wilk test and visually reaffirmed using *QQ* plots. Continuous variables were expressed as mean ± standard deviation for parametric variables or median (interquartile range) for non-parametric variables. Categorical variables were expressed as numbers and percentage. Correlations between PCWP and individual echocardiographic parameters were performed using the Pearson’s two-tailed test. Multiple linear regression models were generated to evaluate independent associations of echocardiographic variables with PCWP. Sensitivity, specificity, negative predictive value (NPV), positive predictive value (PPV), and overall accuracy were measured for each echocardiographic cut-off (as recommended by current guidelines)^2^ using standard definitions. Inter-technique agreement between echocardiographic algorithms and invasive PCWP was tested using calculated *ĸ* coefficients, where 0 to 0.2 was judged as slight; 0.21 to 0.4 as fair; 0.41 to 0.6 as moderate; 0.61 to 0.80 as good, and >0.8 as excellent. Receiver operating characteristics (ROC) analysis was employed to evaluate the discriminative potential of each echocardiographic variable, the ASE/EACVI algorithm, and modified algorithms incorporating LAS_r_ to identify PCWP ≥15 mmHg. Delong’s method^13^ was used to compare area under the curve between algorithms. IBM SPSS statistics version 23.0 was employed for analysis. A *P*-value <0.05 was considered statistically significant.

## Results

### Patient characteristics

**Figure 2:**
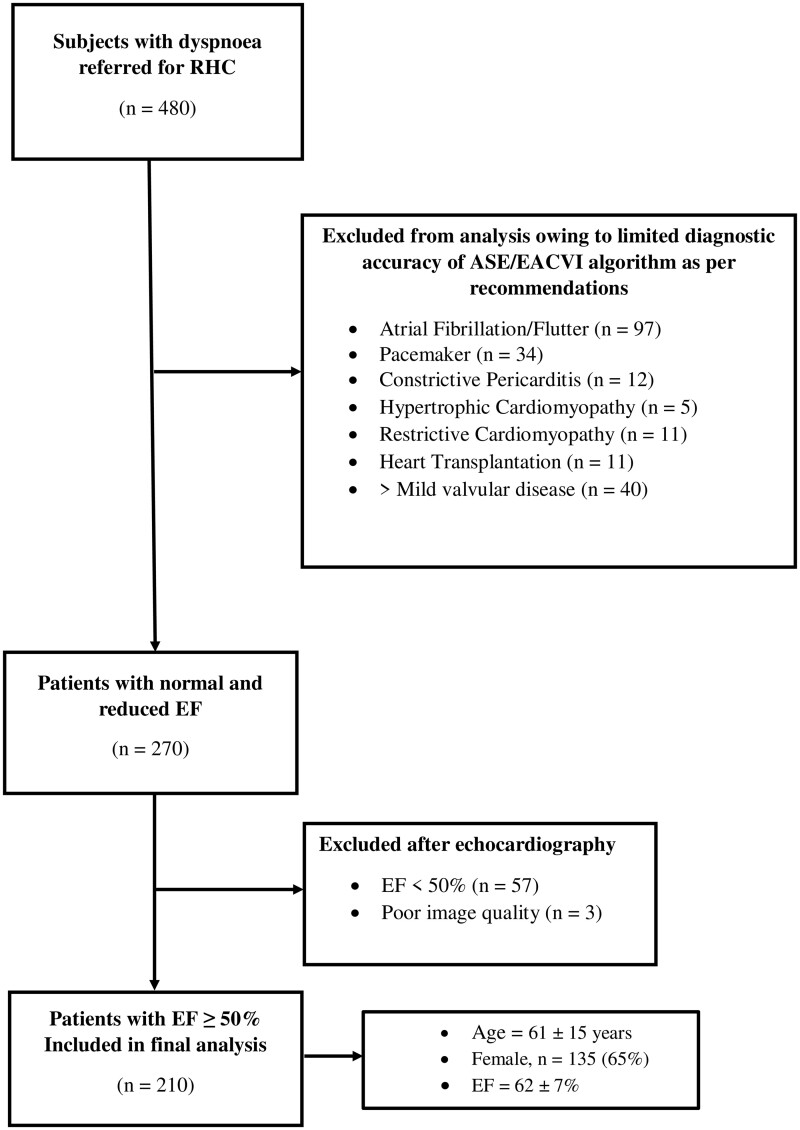
Flow chart of patient selection.

A flow chart of patient selection in our study is presented in *Figure 2*. Of 480 patients referred for catheterization at both centres, 210 met the inclusion criteria. Characteristics of the patient population are presented in *Table 1*. Seventy-four (35%) patients had arterial hypertension, 18 (9%) had diabetes, 38 (18%) had ischaemic heart disease, 77 (37%) had pulmonary hypertension secondary to HF, and 125 patients (60%) had pulmonary parenchymal or vascular disease. Patients with PCWP ≥15 mmHg at rest (*n* = 45; 21%) were older and more frequently diabetic and hypertensive. These patients demonstrated higher right atrial (RA) and pulmonary artery (PA) mean pressures on RHC with lower pulmonary vascular resistance (PVR) than patients with normal PCWP. On echocardiography, these patients demonstrated larger LV volumes and higher LV mass when compared with those with normal filling pressures. EF did not significantly differ between groups (*P* = 0.42). LV global longitudinal strain (LV-GLS) was significantly lower in the group with elevated PCWP (*P* = 0.01). When diastolic variables were compared, patients with elevated PCWP displayed higher mitral *E*-wave velocities, *E*/*A* ratio, *E*/*e*′_mean_ ratio, indexed LA volume, and lower lateral *e*′ velocity (*P* < 0.05 for all comparisons). TR peak velocity and septal *e*′ velocities did not differ between groups (*P* > 0.05 for both comparisons). LAS_r_ was significantly lower in the group with elevated PCWP (16 ± 7 vs. 24 ± 10; *P* < 0.001).

**Table 1:** Clinical characteristics, right heart catheterization, and echocardiographic data of patient population, grouped by PCWP subgroups

	All	PCWP < 15 mmHg	PCWP ≥ 15 mmHg	*P*-value
	(*n* = 210)	(*n* = 165; 79%)	(*n* = 45; 21%)	
Clinical characteristics				
Age (years)	61 ± 15	59 ± 16	68 ± 13	0.001
Female	135 (65%)	110 (66%)	25 (55%)	0.15
Diabetes	17 (8%)	8 (5%)	9 (20%)	0.001
Hypertension	74 (35%)	52 (31%)	22 (49%)	0.02
Ischaemic heart disease	38 (18%)	31 (19%)	7 (16%)	0.58
PH secondary to heart failure	77 (37%)	52 (32%)	25 (56%)	0.001
Pulmonary parenchymal/vascular disease	125 (60%)	108 (65%)	17 (38%)	
Heart rate (bpm)	72 ± 13	73 ± 13	69 ± 14	0.10
Body surface area (m^2^)	1.83 ± 0.22	1.81 ± 0.22	1.91 ± 0.22	0.01
Systolic blood pressure (mmHg)	127 ± 20	125 ± 20	135 ± 21	0.02
Diastolic blood pressure (mmHg)	71 ± 12	71 ± 12	73 ± 13	0.30
NTproBNP (ng/L)	362 (154–1028)	314 (142–778)	500 (316–2113)	0.04
Right heart catheterization				
RAP_mean_ (mmHg)	6 ± 5	5 ± 3	10 ± 6	<0.001
PAP_mean_ (mmHg)	33 ± 14	32 ± 14	38 ± 11	0.002
PCWP (mmHg)	11 ± 5	9 ± 3	19 ± 4	<0.001
TPG (mmHg)	23 ± 14	24 ± 15	19 ± 10	0.03
PVR (WU)	4.8 ± 3.7	5 ± 3.8	4.2 ± 3	0.10
Cardiac output (L/min)	5.1 ± 1.6	5.1 ± 1.5	5.3 ± 1.9	0.50
Echocardiography				
LV end-diastolic volume (mL)	87 ± 35	84 ± 29	101 ± 49	0.004
LV end-systolic volume (mL)	33 ± 22	31 ± 15	40 ± 35	0.01
LVEF (%)	62 ± 7	62 ± 6	61 ± 7	0.42
LV-GLS (%)	17 ± 5	18 ± 5	15 ± 6	0.01
LV mass index (g/m^2^)	87 ± 31	83 ± 29	101 ± 35	0.007
RV basal diameter (mm)	41 ± 8	41 ± 9	42 ± 6	0.24
TAPSE (mm)	19 ± 5	19 ± 5	20 ± 6	0.39
Mitral *E* wave (cm/s)	78 ± 30	72 ± 23	100 ± 40	<0.001
Mitral *E*/*A* ratio	1.2 ± 0.7	1.0 ± 0.4	1.7 ± 1.3	<0.001
Mitral septal *e*′ (cm/s)	6.6 ± 2.6	6.6 ± 2.7	6.6 ± 2.2	0.88
Mitral lateral *e*′ (cm/s)	9.2 ± 3.5	9.4 ± 3.6	8.1 ± 2.7	0.02
Mitral *E*/*e*′_mean_	11 ± 6	10 ± 4	16 ± 9	<0.001
TR peak velocity (m/s)	3.5 ± 0.7	3.5 ± 0.7	3.6 ± 0.6	0.66
LA volume index (mL/m^2^)	30 ± 14	28 ± 12	39 ± 15	<0.001
LA reservoir strain (%)	22 ± 10	24 ± 10	16 ± 7	<0.001

Data are presented as mean ± SD/median (Q1–Q3) or number (%).

EF, ejection fraction; GLS, global longitudinal strain; LA, left atrium; LV, left ventricle; NTproBNP, N-terminal pro-B-type natriuretic peptide; PAP, pulmonary artery pressure; PCWP, pulmonary capillary wedge pressure; PVR, pulmonary vascular resistance; RAP, right atrial pressure; RV, right ventricular; TAPSE, tricuspid annular plane systolic excursion; TPG, transpulmonary gradient; TR, tricuspid regurgitation.

### Correlates of PCWP

**Figure 3:**
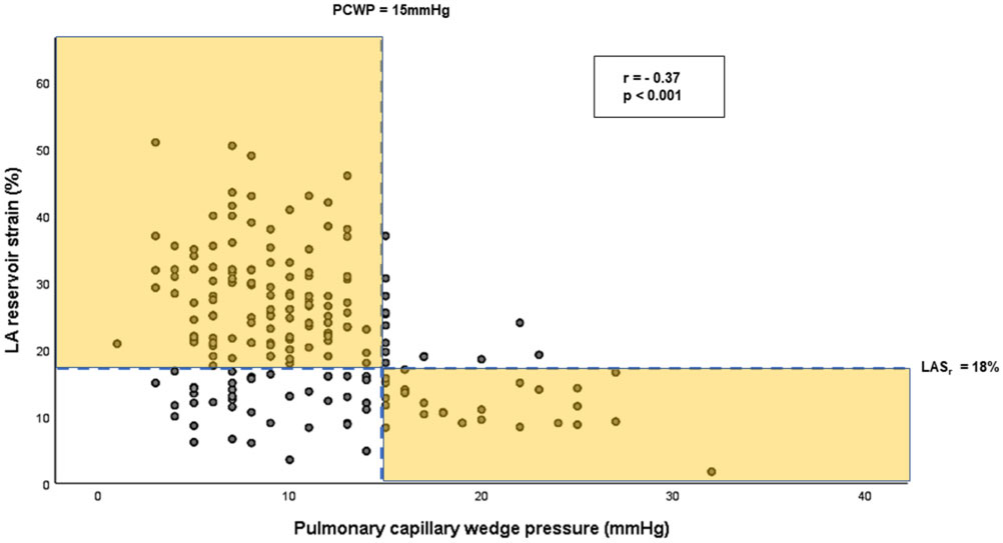
Scatter plot demonstrating relationship between LAS_r_ and PCWP. The highlighted region corresponds with correctly classified LV filling pressure status by LAS_r_ (cut-off 18%).

Correlations of clinical and echocardiographic variables with invasive PCWP are presented in Supplementary data online, *Table S1*. Moderate significant correlations were observed between PCWP and mitral *E*-wave (*r* = 0.50), *E*/*A* ratio (*r* = 0.46), and *E*/*e*′_mean_ ratio (*r* = 0.46, *P* < 0.05 for all). Indexed LA volume demonstrated a mild significant correlation (*r* = 0.36; *P* < 0.001). TR and *e*′ velocities demonstrated no association with PCWP (*P* > 0.05 for all). LAS_r_ demonstrated a significant inverse relationship with PCWP (*r* = −0.37, *P* < 0.001, *Figure 3*) and a positive association with LV-GLS (*r* = 0.33, *P* < 0.001). Multivariable regression analysis was performed to assess independent correlates of PCWP. In the first regression model, we considered mitral *E*-wave, *E*/*A* ratio, *E*/*e*′_mean_ ratio, and indexed LA volume. TR peak velocity, septal, and lateral *e*′ velocities were not considered in the model given absence of significant correlations with PCWP. In this analysis, *E*/*A* (standardized *β* coefficient 0.29; *P* < 0.001) and *E*/*e*′_mean_ ratio (standardized *β* coefficient 0.24; *P* = 0.003) remained correlated with invasive PCWP (*r*^2^ = 0.34; *P* < 0.001). In the second model, when LAS_r_ was considered in addition to the above-listed variables, *E*/*e*′_mean_ was no longer significantly associated with PCWP (*P* = 0.10). Here, mitral *E*-wave (standardized *β* coefficient 0.20; *P* = 0.02), *E*/*A* ratio (standardized *β* coefficient 0.24; *P* < 0.001), and LAS_r_ (standardized *β* coefficient –0.26; *P* < 0.001) emerged as independent variables associated with PCWP (*r*^2^ = 0.41; *P* < 0.001).

### Diagnostic accuracy of echocardiographic variables to identify elevated LV filling pressure

Mitral *E*/*A* ratio ≥2 demonstrated high specificity (96%) and accuracy (80%) to identify elevated PCWP. Sensitivity of *E*/*e*′_mean_, TR velocity, and indexed LA volume recommended cut-offs ranged from 40% to 98%, specificity from 21% to 86%, and accuracy from 38% to 75% (*Table 2*). TR peak velocity >2.8 m/s demonstrated poor specificity (28%) and accuracy (38%) in this analysis. On ROC analysis, mitral *E* wave, *E*/*e*′_mean_, and indexed LA volume demonstrated good ability to identify PCWP ≥15 mmHg (AUC = 0.72; *P* < 0.001 for all three variables). On the other hand, septal *e*′ (AUC = 0.46), lateral *e*′ (AUC = 0.60), and TR peak velocity (AUC = 0.52) demonstrated generally poor ability to identify PCWP ≥15 mmHg (*P* > 0.05 for all variables, Supplementary data online, *Table S2*).

**Table 2:** Sensitivity, specificity, positive predictive value, negative predictive value for echocardiographic cut-offs to identify PCWP ≥15 mmHg

	Cut-off value	Sensitivity (%)	Specificity (%)	PPV (%)	NPV (%)	Accuracy (%)
Mitral *E*/*A* ratio	**≥**2.0	23	96	59	82	80
*e*′ septal	<7 cm/s	44	43	18	73	43
*e*′ lateral	<10 cm/s	67	45	26	83	50
*E*/*e*′_mean_	>14	40	86	44	83	75
LA volume index	>34 mL/m^2^	67	76	44	89	74
LA reservoir strain	<23%	83	53	32	92	59
	<21%	81	64	37	93	67
	<18%	66	72	39	89	71
TR peak velocity	>2.8 m/s	98	21	27	97	38

LA, left atrium; TR, tricuspid regurgitation.

### Diagnostic performance of LAS_r_ to identify elevated PCWP

**Figure 4:**
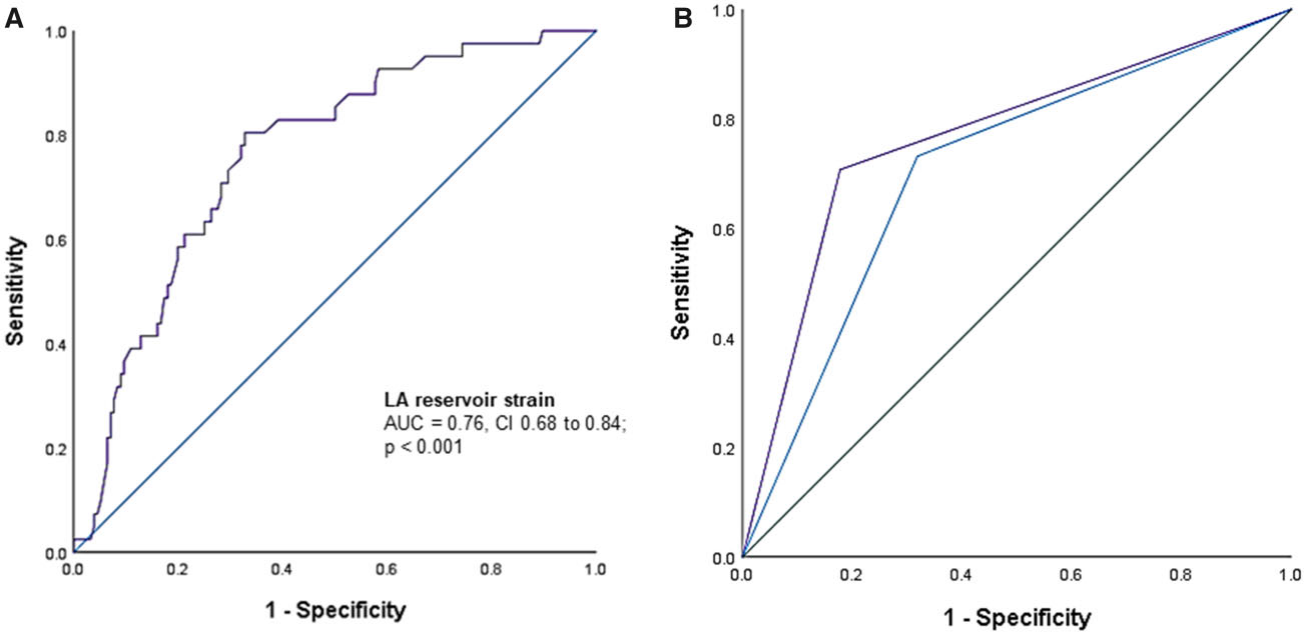
(*A*) Receiver operating characteristic (ROC) analysis demonstrating diagnostic accuracy of LAS_r_ to identify PCWP ≥15 mmHg. (*B*) Receiver operating characteristic (ROC) analysis comparing diagnostic accuracy of ASE/EACVI algorithm (AUC = 0.69) with a modified algorithm that substitutes TR velocity for LAS_r_ to identify PCWP ≥15 mmHg (AUC = 0.77, *P* < 0.05 for comparison).

LAS_r_ demonstrated a strong diagnostic ability to identify elevated PCWP (AUC = 0.76, CI 0.68–0.84; *P* < 0.001, *Figure 4A*). When multiple LAS_r_ cut-offs (23, 21, and 18%) were tested for highest discriminative accuracy, LAS_r_ <21% demonstrated optimally balanced senstitivity and specificity on ROC analysis, displaying 81% sensitivity, 64% specificity, and 67% accuracy to identify invasive PCWP ≥15 mmHg. However, higher overall accuracy (71%) was obtained when LAS_r_ <18% was considered (66% sensitivity, 72% specificity, *Table 2*). Additional sensitivity analysis was performed considering LV-GLS subgroups. In 206 patients where LV-GLS measurements were feasible, 63 (31%) demonstrated LV-GLS <16% and 143 (69%) demonstrated LV-GLS ≥16%. In the LV-GLS ≥16% group, LA reservoir strain at 18% cut-off displayed 65% sensitivity, 84% specificity, and 80% accuracy to determine PCWP ≥15 mmHg (*Table 3*). Supplementary analysis in the lower LV-GLS subgroup demonstrated relatively lower diagnostic performance (Supplementary data online, *Table S3*).

**Table 3:** Sensitivity, specificity, positive predictive value, negative predictive value for LA reservoir strain cut-offs to identify PCWP ≥15 mmHg in patients with left ventricular global longitudinal strain (LV-GLS) ≥16% (*n* = 143; 69%)

	Cut-off value (%)	Sensitivity (%)	Specificity (%)	PPV (%)	NPV (%)	Accuracy (%)
LA reservoir strain	<23	81	64	34	94	67
	<21	77	73	39	93	74
	<18	65	84	47	91	80

### Additional diagnostic contribution of LAS_r_ to ASE/EACVI algorithm

Next, we studied the additional value LAS_r_ adds seperately to Step 1 (that identifies diastolic dysfunction) and Step 2 (that identifies elevated filling pressures) of the conventional diastolic algorithm. Step 1 could be assessed in 175 patients (83%), where all four variables (septal or lateral *e*′, *E*/*e*′, TR velocity, and indexed LA volume) were available. In this analysis, 70 patients (40%) were classified as having normal diastolic function, 60 patients (34%) as indeterminate, and 45 (26%) as having diastolic dysfunction. Among those with indeterminate diastolic dysfunction, LAS_r_ cut-offs identified elevated PCWP with reasonable to good sensitivity (63% to 88% depending on cut-off employed) and modest accuracy (Supplementary data online, *Table S4*). Further, LAS_r_ improved overall accuracy of Step 1 in the ASE/EACVI algorithm, irrespective of cut-off chosen (*Table 4*).

**Table 4:** Sensitivity analysis in Step 1 of the 2-step diastolic dysfunction assessment algorithm to identify PCWP ≥15 mmHg

Diastolic algorithm	Sensitivity (%)	Specificity (%)	PPV (%)	NPV (%)	Accuracy (%)
ASE/EACVI algorithm	83	47	32	90	55
ASE/EACVI algorithm adding LAS_r_ (<23%)	79	65	40	91	68
ASE/EACVI algorithm adding LAS_r_ (<21%)	76	68	41	91	70
ASE/EACVI algorithm adding LAS_r_ (<18%)	68	69	39	88	69

Estimation of LV filling pressures employing Step 2 of the ASE/EACVI algorithm was feasible in 187 patients (89%). Assessment of transmitral *E* velocity and mitral *E*/*A* ratio classified 26 patients (12%) as having normal, 167 (80%) as requiring additional criteria (*E*/*e*′_mean_, TR velocity, and indexed LA volume) and 17 (8%) as having elevated LV filling pressures. When additional parameters were also assessed, Step 2 of the ASE/EACVI algorithm classified 110 patients (52%) as having normal LV filling pressures, 23 patients (11%) as having indeterminate filling pressures, and 77 patients (37%) having elevated filling pressures. Step 2 of the ASE/EACVI algorithm demonstrated 71% sensitivity, 68% specificity, 68% accuracy, and -reasonable discriminative ability to identify elevated PCWP (AUC = 0.69, CI 0.60–0.78; *P* < 0.001, *Table 5*).

**Table 5:** Feasibility and diagnostic performance of different approaches or algorithms to determine elevated LV filling pressure (PCWP ≥ 15 mmHg) in patients with preserved LVEF

Diastolic algorithm	Feasibility (%)	Sens (%)	Spec (%)	PPV (%)	NPV (%)	Accuracy (%)	AUC	CI	*P*-value
ASE/EACVI algorithm	89	71	68	40	88	68	0.69	0.60–0.78	<0.001
Model 1: ASE/EACVI algorithm substituting TR velocity for LAS_r_ (cut-off 18%)	91	69	84	55	91	81	0.77	0.67–0.85	<0.001
Model 2: Algorithm based on EACVI recommendations for multimodality imaging in HFpEF, replacing any missing additional parameter with LAS_r_ (cut-off 18%)^9^	98	71	70	40	90	70	0.71	0.62–0.79	<0.001
Model 3: ASE/EACVI algorithm adding LAS_r_ (cut-off 18%) to conventional variables	90	67	84	56	89	80	0.75	0.66–0.84	<0.001

**Figure 5:**
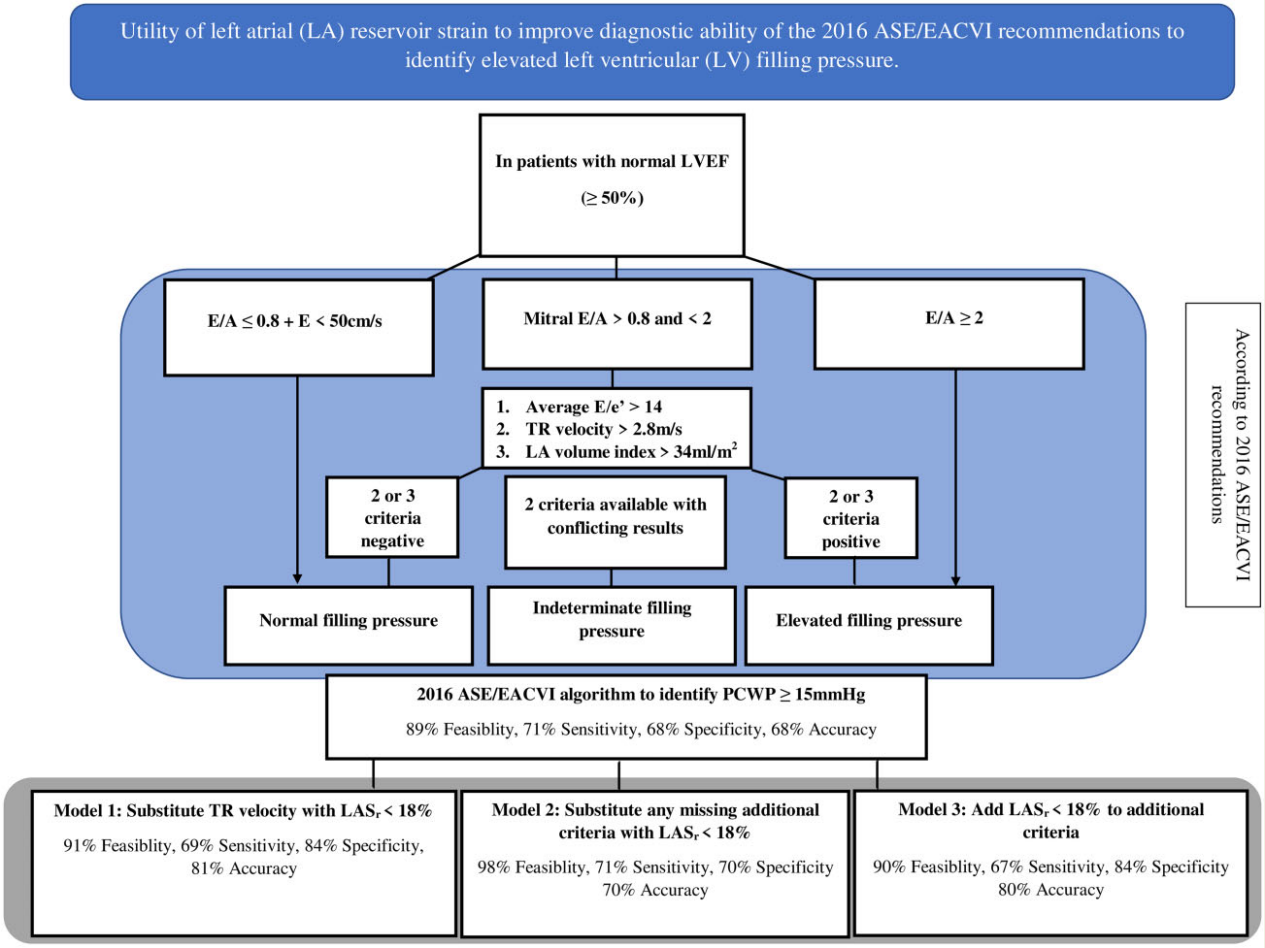
Models displaying the utility of left atrial (LA) reservoir strain to improve diagnostic ability of the 2016 ASE/EACVI recommendations to identify elevated left ventricular (LV) filling pressure.

We studied the potential value LAS_r_ adds to the conventional diastolic algorithm to assess LV filling pressures using three different models. Salient findings of this analysis have been presented in *Figure 5* and the *Graphical Abstract*. In Model 1, we substituted TR peak velocity >2.8 m/s (which demonstrated lowest feasibility and poor overall accuracy to detect elevated filling pressures) with LAS_r_ <18%. This approach (sensitivity 69%, specificity 84%) demonstrated higher feasibility (91% vs. 89%), overall accuracy (81% vs. 68%), and greater agreement with invasive measurements (*ĸ* coefficient 0.48 vs. 0.30) when compared with the ASE/EACVI approach (*Table 5*). Comparison of ROC curves demonstrated higher diagnostic performance to identify elevated filling pressures using Model 1 when compared with the conventional algorithm (AUC = 0.77 vs. 0.69, *P* = 0.001). In Model 2, in keeping with the EACVI recommendations for multimodality imaging in HFpEF,^9^ when one of the additional variables (*E*/*e*′, TR velocity, or indexed LA volume) were missing and the other two were conflicting, the missing variable was replaced with LAS_r_. This approach (sensitivity 71%, specificity 70%) demonstrated higher feasibility than the ASE/EACVI algorithm (98% vs. 89%), in addition to marginally higher specificity (70% vs. 68%), accuracy (70% vs. 68%), and greater agreement with invasive measurements (*ĸ* coefficient 0.32 vs. 0.30). Finally, in Model 3, we considered LAS_r_ in addition to conventional parameters used in the ASE/EACVI algorithm. To clarify, after first evaluating filling pressures based on transmitral velocities and *E*/*A* ratio, we added LAS_r_ to the additional criteria (*E*/*e*′, TR velocity, and indexed LA volume). Using this approach, an elevated filling pressure status was assigned if >50% of the additional criteria (3 or 4) were found positive, and non-elevated if ≤50% (1 or 2) were found positive. This approach also demonstrated higher specificity (84% vs. 68%), accuracy (80% vs. 68%), agreement with invasive assessment (*ĸ* coefficient 0.47 vs. 0.30), and a tendency to higher diagnostic performance on ROC analysis (AUC = 0.75 vs. 0.69; *P* = 0.06) when compared with the ASE/EACVI algorithm (*Table 5*).

### Feasibility and reproducibility

Measurement of echocardiographic surrogates of diastolic dysfunction in the ASE/EACVI algorithm were highly feasible (Supplementary data online, *Table S2*). TR peak velocity could be measured in 89% of cases. Measurement of LAS_r_ could be performed in 94% of patients. Double measurements in 40 randomly selected patients demonstrated a coefficient of variation of 10% with an intra-class correlation coefficient of 0.91 (95% CI 0.73–0.96). Test–retest analysis for LAS_r_ yielded a coefficient of variation of 12.8%.

## Discussion

In a large, dual-centre, haemodynamic database of patients with unexplained dyspnoea and preserved EF, LAS_r_ was independently associated with invasive PCWP and demonstrated strong diagnostic ability to identify elevated LV filling pressure. Applying LAS_r_ to the 2016 ASE/EACVI diastolic algorithm enhanced feasibility of the recommended approach, in addition to overall accuracy and agreement with invasive filling pressure. Our findings support an important role for LAS_r_ in routine echocardiographic assessment of LV filling pressure status.

### Diagnostic performance of ASE/EACVI guidelines

Since their introduction in 2016, the ASE/EACVI recommendations for evaluation of diastolic function have been validated in multiple cohorts but have shown conflicting results. Initial studies performed in patients with a wide spectrum of cardiac disease including both reduced and preserved EF suggest generally good diagnostic performance.^14–17^ Most of these studies included patients with suspected coronary artery disease referred for left heart catheterization^14,^^17^ and, by design, excluded patients with pulmonary disease. Further, even in these studies, a generally poorer sensitivity was observed when patients with preserved EF were exclusively considered.^14^ Recently, in a smaller cohort (*n* = 63) of patients with pulmonary hypertension of which 44% had left heart disease, the ASE/EACVI algorithm demonstrated high sensitivity and specificity (84% and 80%, respectively) to identify elevated filling pressures, defined as PCWP >12 mmHg.^18^ The study, however, did not exclude all cardiac conditions where recommendations suggest that the ASE/EACVI algorithm is less reliable^2^ and this, in addition to a lowered PCWP cut-off, may have influenced sensitivity analysis. We chose to strictly mirror selection to exclude challenging sub-populations in keeping with recommendations.^2^ Despite careful patient selection, *e*′ (irrespective of whether septal or lateral velocities were considered) and TR peak velocity demonstrated no significant association with invasive PCWP and poor ability to distinguish elevated filling pressure in our cohort. Lack of association of these specific variables with PCWP has been recently reported in another stringently selected cohort and may be attributed to the inclusion of patients with pulmonary disorders in both our and the above-mentioned cohort.^6^ Further, *E*/*e*′, which is widely utilized and has the most evidence as per recent systematic reviews^3^ demonstrated only a modest correlation with PCWP and showed no significant association with PCWP when LAS_r_ was introduced into our multivariable regression model.

Differentiating HFpEF from pulmonary disease is a common clinical conundrum in patients presenting with unexplained breathlessness. While our findings may not be applicable to a general HFpEF population, they are consistent with what is seen in clinical practice at specialist PH centres and provide a real-world context. Furthermore, our findings are consistent with other studies in patients with preserved EF that suggest that individual variables demonstrate poor ability to represent invasive pressures, and advocate a multi-parametric approach to filling pressure assessment.^3,^^5,^^6^

### Incremental value of LAS_r_ to filling pressure assessment

Studies showcasing modest ASE/EACVI algorithm discriminatory performance suggest that complementary scores and/or more advanced investigative approaches may improve diagnostic accuracy and reduce indeterminate classification.^6^ In a recent EACVI survey to study adoption of the ASE/EACVI guidelines in routine practice, LAS_r_ was chosen by 34% of respondents as a method of choice in challenging diagnostic scenarios.^19^ Additionally, LAS_r_ has been incorporated in a revised algorithm in the recent EACVI recommendations for multimodality imaging in HFpEF.^9^ LA phasic function can be evaluated during the reservoir, conduit, or contractile phase and changes in strain has been shown to be independent of LA volume in HFpEF.^20^ We chose to evaluate deformation during the reservoir phase, LAS_r_, based on high feasibility, wider utilization, and greater agreement with worsening diastolic dysfunction when compared with other LA phasic strain measures.^7^ LAS_r_ is reduced in diastolic dysfunction, demonstrates better agreement with invasive LV filling pressure when compared with conventional algorithms when EF is preserved,^21^ and accurately grades diastolic dysfunction severity.^7^ Further, LAS_r_ has previously been shown to correlate well with elevated filling pressure in HF during rest,^22^ and has demonstrated strong ability to discern disproportional pressure rise during exercise.^23^ Our data suggest that incorporating LAS_r_ into the current diastolic algorithm further enhances its feasibility, accuracy, and diagnostic performance and, hence, may be a promising clinical tool. Enhanced accuracy seen in models considering LAS_r_ in this study was driven predominantly by improved specificity of the revised approaches to determine elevated filling pressures. The stronger ability of these modified echocardiographic approaches to ‘rule-in’ diastolic dysfunction by better identification of those with non-elevated filling pressure may further enhance utility to screen and triage patients with unexplained breathlessness. Given that LA filling corresponds with LV long-axis shortening during systole, further studies are necessary to establish additional information LAS_r_ provides over what LV longitudinal strain already offers.

### Clinical relevance

Our study has important clinical implications in the management of unexplained dyspnoea, as accurate identification of the underlying cause has direct consequences on choice of therapy. This is even more relevant in the setting of normal EF, where focused assessment of LV size and systolic function may not suitably explain the patient’s symptoms. While the accuracy of LA pump strain to identify normal filling pressure in preserved EF has recently been suggested,^8^ our data add that LAS_r_ may also offer additional value when complementing the 2016 ASE/EACVI recommendation as suggested recently by the EACVI recommendations for multimodality imaging in HFpEF.^9^

### Limitations

Important limitations of the current study are the low proportion of patients with elevated PCWP and inclusion of patients with pulmonary parenchymal or vascular disorders. This may bias our results, which include sensitivity analysis in the proposed models incorporating LAS_r_. Further validation of these findings is necessary in additional large-sample, multicentre studies. Our study cohort comprised patients with unexplained breathlessness referred to two tertiary-care centres and may not reflect a general HFpEF population. Nevertheless, the study presents a real-world scenario in PH specialist centres where distinction between HFpEF and pulmonary disorders is often challenging and stronger screening algorithms to rule-in diastolic dysfunction are needed. We employed fluid-filled catheters to obtain PCWP measurements rather than high-fidelity microcatheters and this may be considered a limitation. Inter-operator and inter-evaluator variability may be considered a limitation in our study, given that we did not employ a core-lab approach to echocardiographic image analysis. However, a standard international acquisition and analysis protocol was followed by two experienced echocardiographers with over 15 years’ experience. Further, we evaluated the contribution of LAS_r_ to diastolic assessment in the same cohort rather than employ a retrospective derivation and independent prospective validation cohort, which may have strengthened our study design. Absence of information on LA pump strain in this cohort may also be considered a limitation, given that high LA pump strain has demonstrated strong ability to identify patients with normal filling pressure when EF is preserved.^8^ However, we chose to focus on LAS_r_ which is more robust, and in keeping with the recent expert consensus algorithm.^9^ Finally, diastolic stress tests were not performed in all our patients. Although stress testing may have improved the diagnostic accuracy of the ASE/EACVI algorithm, this was not the aim of this study.

## Conclusion

In the setting of preserved EF, incorporation of LAS_r_ into the 2016 ASE/EACVI recommendation-based assessment improves detection of elevated filling pressure.

## Supplementary data

Supplementary data are available at *European Heart Journal - Cardiovascular Imaging* online.

## Funding

A.V. was supported by grants from the Swedish Association for Pulmonary Hypertension; L.H.L. was supported by grants from the Swedish Research Council (2013-23897-104604-23 and 523-2014-2336), Swedish Heart Lung Foundation (20100419 and 20120321), Stockholm County Council (20110120 and 20140220), and Swedish Society of Medicine (174111 and 504881). P.L. was supported by grants from The Swedish Heart and Lung foundation (20160787 and 20200160) and the Swedish Research Council (2019-01338).

## Supplementary Material

jeac036_Supplementary_Data

## Data Availability

The data underlying this article cannot be shared publicly due to the privacy of individuals that participated in the study and GDPR regulations.
