# Supplementary material for: Left atrial reservoir strain improves diagnostic accuracy of the 2016 ASE/EACVI diastolic algorithm in patients with preserved left ventricular ejection fraction: insights from the KARUM haemodynamic database
Source: Eur Heart J Cardiovasc Imaging. 2022 Feb 19;23(9):1157–68. doi: 10.1093/ehjci/jeac036 (PMC9635061; doi:10.1093/ehjci/jeac036)
Supplement: jeac036_Supplementary_Data [file jeac036_supplementary_data.docx]

**Supplementary Table 1.** Pearson’s correlation between PCWP and selected patient characteristics

| **Parameters** | **r-value** | **P-value** |
| --- | --- | --- |
| Age | 0.24 | <0.001 |
| Heart rate | -0.15 | 0.02 |
| Systolic blood pressure | 0.21 | 0.002 |
| Diastolic blood pressure | 0.03 | 0.60 |
| Body surface area | 0.20 | 0.003 |
| NTproBNP | 0.30 | <0.003 |
| LV end-diastolic volume | 0.24 | <0.001 |
| LV end-systolic volume | 0.25 | <0.001 |
| LV mass index | 0.28 | 0.001 |
| LVEF | -0.07 | 0.25 |
| LV global longitudinal strain | -0.15 | 0.02 |
| Mitral E-wave | 0.50 | <0.001 |
| Mitral E/A ratio | 0.46 | <0.001 |
| Mitral septal e’ | 0.03 | 0.69 |
| Mitral lateral e’ | -0.13 | 0.06 |
| Mitral E/e’_mean_ | 0.46 | <0.001 |
| TR peak velocity | 0.03 | 0.66 |
| LA volume index | 0.36 | <0.001 |
| LA reservoir strain | -0.37 | <0.001 |

NTproBNP, N-terminal pro-B-type natriuretic peptide; LV, left ventricle; EF, ejection fraction; GLS, global longitudinal strain; TR, tricuspid regurgitation; LA, left atrium.

**Supplementary Table 2.** Feasibility and diagnostic ability of echocardiographic variables to identify PCWP ≥ 15mmHg in final patient cohort (n = 210)

|  | **Feasibility** | **AUC** | **CI** | **P-value** |
| --- | --- | --- | --- | --- |
| Mitral E-wave | 100% | 0.72 | 0.62 to 0.81 | <0.001 |
| Mitral E/A ratio | 98% | 0.68 | 0.58 to 0.78 | <0.001 |
| e’ septal | 93% | 0.46 | 0.37 to 0.55 | 0.44 |
| e’ lateral | 94% | 0.60 | 0.51 to 0.69 | 0.05 |
| E/e’_mean_ | 97% | 0.72 | 0.63 to 0.80 | <0.001 |
| LA volume index | 98% | 0.72 | 0.63 to 0.81 | <0.001 |
| TR peak velocity | 89% | 0.52 | 0.43 to 0.61 | 0.66 |
| LA reservoir strain | 95% | 0.76 | 0.68 to 0.84 | <0.001 |

LA, left atrium; TR, tricuspid regurgitation.

|  | **Cut-off value** | **Sensitivity** | **Specificity** | **Positive predictive value** | **Negative predictive value** | **Accuracy** |
| --- | --- | --- | --- | --- | --- | --- |
| LA reservoir strain | <23% | 87% | 22% | 29% | 82% | 39% |
|  | <21% | 87% | 37% | 33% | 88% | 50% |
|  | <18% | 67% | 42% | 29% | 77% | 48% |

**Supplementary Table 3.** Sensitivity, specificity, positive predictive value, negative predictive value for LA reservoir strain cut-offs to identify PCWP ≥ 15mmHg in patients with left ventricular global longitudinal strain (LV-GLS) < 16% (n = 63; 31%)

**Supplementary Table 4**. Rate of PCWP < and ≥ 15mmHg for LA reservoir cut-offs 23%, 21% and 18% in the indeterminate diastolic dysfunction group (n = 60) identified using Step 1 of the ASE/EACVI diastolic algorithm.

|  | **PCWP < 15mmHg** | **PCWP ≥ 15mmHg** |
| --- | --- | --- |
| **LASr < 23%** | 21 | 14  (Sensitivity 88%) |
| **LAS_r_ ≥ 23%** | 23  (Specificity 52%) | 2 |

Accuracy 62%

|  | **PCWP < 15mmHg** | **PCWP ≥ 15mmHg** |
| --- | --- | --- |
| **LASr < 21%** | 17 | 13  (Sensitivity 81%) |
| **LAS_r_ ≥ 21%** | 27  (Specificity 61%) | 3 |

|  | **PCWP < 15mmHg** | **PCWP ≥ 15mmHg** |
| --- | --- | --- |
| **LASr < 18%** | 16 | 10  (Sensitivity 63%) |
| **LAS_r_ ≥ 18%** | 28  (Specificity 64%) | 6 |

Accuracy 67%

Accuracy 63%
